# High Content Solid Dispersions for Dose Window Extension: A Basis for Design Flexibility in Fused Deposition Modelling

**DOI:** 10.1007/s11095-019-2720-6

**Published:** 2019-12-17

**Authors:** Rydvikha Govender, Susanna Abrahmsén-Alami, Staffan Folestad, Anette Larsson

**Affiliations:** 1 Pharmaceutical Technology and Development, AstraZeneca Gothenburg, Pepparedsleden 1, 43183 Mölndal, Sweden; 2 Chemistry and Chemical Engineering, Chalmers University of Technology, Gothenburg, Sweden

**Keywords:** dose flexibility, fused deposition modelling, modular design, solid dispersion, uniformity of drug content

## Abstract

**Purpose:**

This study uses high drug content solid dispersions for dose window extension beyond current demonstrations using fused deposition modelling (FDM) to; i) accommodate pharmaceutically relevant doses of drugs of varying potencies at acceptable dosage form sizes and ii) enable enhanced dose flexibility via modular dosage form design concepts.

**Methods:**

FDM was used to generate ~0.5 mm thick discs of varying diameter (2–10 mm) from melt-extruded feedstocks based on 10% to 50% w/w felodipine in ethyl cellulose. Drug content was determined by UV spectroscopy and dispensing precision from printed disc mass.

**Results:**

Mean felodipine content was within ±5% of target values for all print volumes and compositions including contents as high as ~50% w/w. However, poor dispensing precision was evident at all print volumes.

**Conclusions:**

In pursuit of dose flexibility, this successful demonstration of dose window extension using high content solid dispersions preserves FDM design flexibility by maintaining applicability to drugs of varying potencies. The achieved uniformity of content supports the application of varying content solid dispersions to modular dosage form concepts to enhance dose flexibility. However, poor dispensing precision impedes its utilisation until appropriate compatibility between FDM hardware and materials at varying drug contents can be attained.

## Introduction

Fused deposition modelling (FDM) is an additive manufacturing (AM) technology based on material extrusion. Specifically, FDM involves feeding of a thermoplastic polymeric filament through a heated nozzle where it melts or softens for subsequent layer-by-layer deposition on a platform (1). Like AM technologies in general, the versatility, freedom of design and formation of complex, customizable parts makes FDM attractive for pharmaceutical applications (2), particularly for the generation of individualized products (3–6). FDM is preceded by hot melt extrusion (HME) to develop a filament feedstock for the FDM process. HME, involving the mixing of materials by rotating screws under elevated temperature and shear, is widely used for the formation of solid dispersions of drugs in polymeric carriers (7,8). It is claimed to enable homogeneous drug distribution particularly when solid solutions (molecular dispersions) of a drug in the polymeric carrier are formed (6,8–10). This provides a distinct benefit for flexible dosing. Patients’ needs for flexible dosing are ascertained from multiple characteristics (*e.g.* genetics, age, body weight, disease severity, comorbidities, adherence, food-drug and drug-drug interactions), which vary between individuals and within the same individual over time (11–16). Tailoring the dose to these characteristics to promote safety and effectiveness necessitates the provision of an increased number of dose strengths in the product offering (17).

Dose flexibility has been demonstrated previously using the current state of the art in FDM. One approach used to provide flexibility is alteration of dosage form size to accommodate varying dose strengths (18–20). Drug loading has, until now, been based on a filament of low drug content, for example 2% w/w (21) and as low as 0.29% w/w (1) for drug loading methods based on passive diffusion of drug from solution into commercially available pre-extruded filaments of high diameter tolerances. Consequently, dose ranges that have been demonstrated so far, for conventional FDM, are often insufficient to incorporate pharmaceutically relevant doses of intermediate to low potency drugs without resulting in unacceptably large dosage forms. Although lab-scale compounding of filaments by HME from physical mixtures of drug and carrier broadens the scope of materials that can be used and enables improved drug loading, co-processing the drug and polymer may introduce variations in rheological properties and filament diameter (18,22). This not only has implications for FDM printability and repeatability downstream but has limited the study of high drug content compositions in current literature. Few studies have attempted to vary the dose independent of dosage form size *e.g.* by altering the infill percentage of printed units (1), however, these are still based on low payload filaments.

To the best of our knowledge, size-independent dose flexibility at pharmaceutically relevant doses has not yet been demonstrated with conventional FDM and has instead required modification of printer hardware *e.g.* co-ordinated 3D printing and liquid dispensing (23). In addition to a limited dose range achievable with the dose-dependent dosage form size approach, the extent of flexibility, i.e. the number of dose increments within the dose range, is directly dependent on the number of discrete filament compositions that can be produced.

**Fig. 1:**
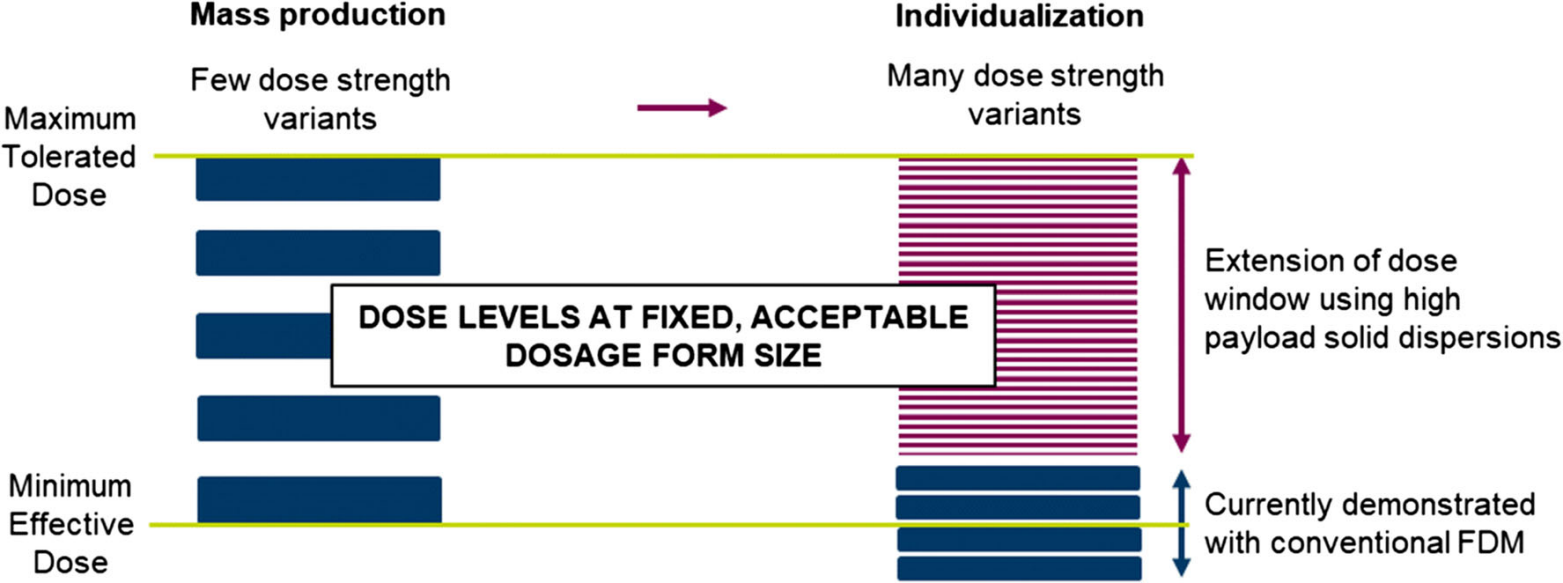
Enabling dose flexibility for individualization by use of high drug content solid dispersions in FDM for dose window extension relative to current demonstrations.

In response to the aforementioned drawbacks in current attempts at dose flexibility using FDM, we suggest the use of high drug content solid dispersions to extend the dose window to accommodate pharmaceutically relevant doses of drugs of varying potencies at acceptable dosage form sizes (Fig. 1).

Our suggested approach is derived from the following hypothesis: Uniformity of drug content in printed units, a prerequisite for dose flexibility, can be concurrently achieved at high feedstock drug contents and small print volumes, provided that homogeneity is maintained at an equivalent or smaller length scale in the feedstock than the size of a printed unit.

In most cases, drug-containing FDM-printed units encountered in the current literature have dimensions in the same order of magnitude as conventional tablets (1,19,20,24,25). For a standard size dosage form, a greater degree of dose flexibility can be achieved if the dosage form is comprised of smaller modules, *e.g.* pellets (26). This will entail printing of smaller volumes of each module than the size of a final dosage form. Our study therefore aims to investigate uniformity of drug content at 10, 30 and 50% w/w API and at small print volumes ranging from ~2 to ~40 mm^3^.

A model system comprising felodipine (FEL) as the model drug and ethyl cellulose (EC) as the polymeric carrier was selected based on an inherently low tendency of FEL to recrystallize from the amorphous form (27), which is further promoted by the ability of EC to stabilize FEL in amorphous form (28,29). This system was postulated to form sufficiently stable amorphous solid dispersions at high drug contents without a tendency to introduce large domain inhomogeneity via recrystallization or amorphous phase separation prior to FDM, allowing investigation of the study aims.

## Materials and Methods

### Materials

FEL (MW 384.26 g/mol) was obtained from AstraZeneca, Sweden. EC (Ethocel™ Standard 20 Premium) was supplied by Dow (Dow Europe GmbH, Sweden). Ethanol (95%) was analytical grade.

### Methods

#### Material Thermal Characterisation

The onset of thermal degradation (T_deg_) was determined by thermogravimetric analysis (TGA) using a TGA/DSC 3^+^ STAR^e^ system instrument (Mettler Toledo, Switzerland). T_deg_ was calculated as the extrapolated onset temperature in the weight *vs.* temperature curve. FEL and EC powders were weighed in open 70 μl alumina crucibles and heated from 25°C to 350°C at 10 K/min under a nitrogen atmosphere set at a flow rate of 50 ml/min.

Differential scanning calorimetry (DSC) in a DSC 2 STAR^e^ system instrument (Mettler Toledo, Switzerland) was used to measure both the glass transition temperatures (T_g_) and melting points (T_m_) of FEL and EC powders as well as HME filaments after 24 h storage in sealed plastic bags at ambient conditions. T_g_ values were determined at the midpoint of the T_g_ range and T_m_ at the peak of the melting endotherm. Each sample was weighed in a 40 μl aluminium crucible, which was sealed by a lid with pinhole for subsequent analysis. The instrument was run in a heat-cool-heat cycle at 10 K/min from 25°C to 210°C under a nitrogen atmosphere with a flow rate of 50 ml/min. STAR^e^ software (version 16.00b, Mettler Toledo, Switzerland) was used for instrument control and subsequent analysis of thermograms.

#### HME

Three compositions were prepared corresponding to 10% w/w, 30% w/w, and 50% w/w FEL in EC, which are designated FEL10, FEL30, and FEL50, respectively. FEL10HME, FEL30HME, and FEL50HME represent hot melt extruded filaments at each FEL concentration. EC powder and FEL powder were weighed in glass vials in approximately 2.5 g batches and mixed until homogeneous upon visual inspection. HME was performed using a 5 ml capacity Xplore micro compounder (Xplore, The Netherlands), affixed with conical mixing screws and a circular die 1.5 mm in diameter. The physical mixtures were fed via a hopper into the barrel maintained at a constant temperature profile of 150°C and a screw speed of 50 rpm during feeding, recirculation and ejection, for all compositions. After complete feeding (˂ 1 min), melted mixtures were recirculated for 10 min to aid homogenization prior to manual extrusion through the die to obtain a cylindrical filament, which was allowed to cool at ambient temperature. After extrusion, the length of each filament was measured and the diameter was verified every 5 cm using digital calipers. Filaments were stored in sealed plastic bags at ambient temperature prior to characterisation or processing by FDM.

#### FDM

A ZMorph VX multitool 3D printer (ZMorph S.A., Wroclaw, Poland), equipped with a single 0.3 mm diameter extrusion nozzle, was used to print disc-shaped units according to a digital model created using Autodesk® TinkerCAD™ (Autodesk, Inc., San Rafael, CA, USA). Discs were designed in 5 diameter variants including 2, 4, 6, 8, and 10 mm, all 0.5 mm thick. Calculated target volume for each variant was 1.6, 6.3, 14.1, 25.1, and 39.3 mm^3^, respectively. The generated ‘.stl’ file was subsequently imported into Simplify3D® (version 4.1.1., Simplify3D LLC) for control of printing parameters and subsequent printing. The build platform was levelled and heated to 80°C prior to printing of discs. After 24 h of storage at ambient temperature, prepared filaments were manually fed into a 0.3 mm nozzle onto the heated glass build platform overlaid with a polyethylene terephthalate (PET) film to facilitate adhesion and aid detachment of thin discs upon cooling. Due to filament breakage when inserted into a filament feeding mechanism based on rotating gears, automated feeding was not achievable. Nozzle temperature was adjusted to 195°C, 170°C, and 155°C for FEL10, FEL30, and FEL50, respectively, ensuring suitable melt flow for printability. FEL10FDM, FEL30FDM, and FEL50FDM represent FDM-printed discs at each FEL content.

#### FEL Assay in HME Filaments and FDM Discs

Ten 0.5 cm samples were obtained from each filament at a minimum of 5 cm intervals to allow sample collection over the length of the entire extruded filament, with longer intervals for longer filaments. The first 2.5 cm, corresponding to the extruder die length, was excluded. A total of 60 samples were obtained from 2 filaments per composition. Each 0.5 cm filament section was weighed with a Mettler MT5 analytical balance (Mettler Toledo, Switzerland) and dissolved, whilst stirring, in 10 ml 95% ethanol at ambient temperature for 24 h.

Discs generated by FDM were weighed and dissolved as for HME filaments but solvent volumes for dissolution of discs were adapted to disc size (5 ml for 2 mm discs, 10 ml for 4–8 mm discs and 20 ml for 10 mm discs) to allow solvent volumes sufficiently above the solubility limit of FEL and EC in ethanol. Disc duplicates (2 mm to 10 mm) were printed from each of two filaments per composition resulting in a total of 30 discs for FEL assay.

Amber glassware was used for all analyses. FEL content was determined by analysing sample solutions with a Cary60 UV-Vis spectrophotometer (Agilent Technologies, Inc., CA, USA). Cary WinUV scan application software (version 5.0.0.999, Agilent Technologies, Inc., CA, USA) was used for instrument operation and spectral acquisition. Sample solutions were analysed at a scan rate of 4800 nm/min from 800 to 290 nm, excluding lower wavelengths due to degradation of FEL at these wavelengths. Absorbance readings at a lambda max of 362 nm were used to calculate FEL content. Linearity was confirmed by a calibration curve of FEL in 95% ethanol over the range 5 to 45 μg/ml. Absence of interaction between FEL and EC in solution was confirmed by UV absorbance measurements of a 1:1 mixture of FEL and EC solutions at 20 μg/ml. Prior to analysis, sample solutions were diluted, as required, in 95% ethanol to fall within this range. UV absorbance measurements were performed in triplicate for each sample solution.

#### Fourier Transform Raman Spectroscopy

Fourier transform Raman spectroscopy (FT-Raman spectroscopy) was performed on raw materials and extruded filaments (FEL10HME, FEL30HME, and FEL50HME). A MultiRAM FT-Raman spectrometer (Bruker OPTIK GmbH, Ettlingen, Germany) was used, equipped with an Nd:YAG laser excitation source operating at 1064 nm. OPUS Version 7.5 (Bruker OPTIK GmbH, Ettlingen, Germany) was used for instrument operation and data acquisition. Spectra were recorded as an average of 256 scans for each sample at a laser power of 500 mW to obtain a good signal-to-noise ratio at a resolution of 2 cm^−1^. FEL and EC powders were placed in nuclear magnetic resonance (NMR) tubes and filament sections obtained mid-length from the total extruded length were mounted directly on a sample holder before placing in the optical path of the spectrometer. For the amorphous FEL reference, FEL powder was placed in an NMR tube and heated in a vacuum oven at 150°C until FEL powder was melted.

## Results

### Uniformity of Drug Content

**Fig. 2:**
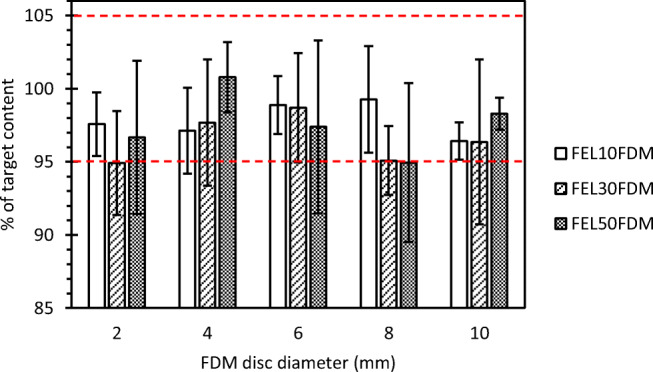
Mean FEL content as a % of target FEL content in FDM discs at each composition and disc size; each bar = mean ± SD of *n* = 4 discs.

Figure 2 shows FEL content as a % of target content in printed discs after normalizing to mass, where target content was defined as 8.8% w/w, 25.9% w/w, and 42.8% w/w FEL for FEL10, FEL30, and FEL50, respectively, denoting filament FEL content after the corresponding extrusion step. Mean FEL content, to the nearest whole number, was within 5% of target values and individual FEL contents were within 10% of target values at all drug contents and all print volumes, even for the high FEL content composition (FEL50FDM).

**Fig. 3:**
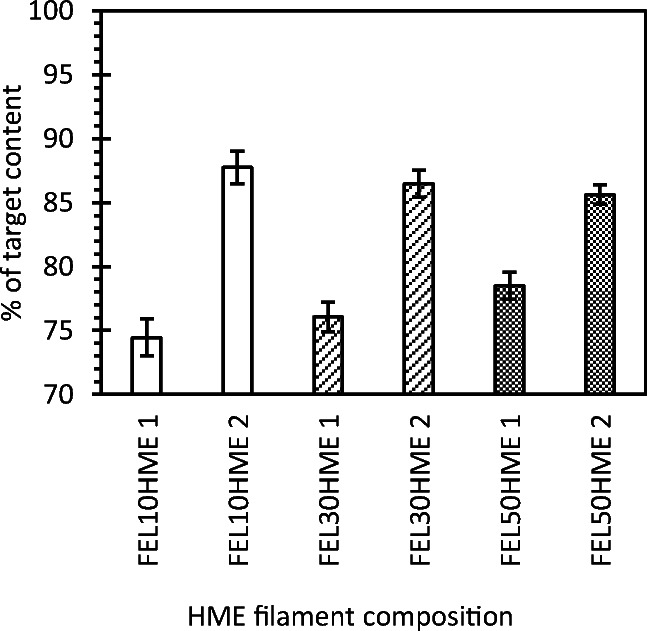
Mean FEL content as a % of target FEL content in HME filaments at each composition; 1 and 2 denote sequential extrusion steps; each bar = mean ± SD of *n* = 10 sections from the same filament.

The tendency of drugs to aggregate typically increases with concentration, potentially impacting both stability against recrystallization and homogeneity. Regarding homogeneity, this is therefore a critical finding since the use of high content solid dispersions is a prerequisite for achieving dose flexibility at pharmaceutically relevant doses. To this end, achieving an accurate and precise target dose in printed discs at predicted print volumes ranging from 1.5 to 39 mm^3^ was hypothesized to be dependent on homogeneity in the filament extrudate on an equivalent or smaller scale than the desired print volume. Indeed, the FEL content in sections of extruded filaments spanning the full length of each filament at sample volumes ~ 0.9 mm^3^ revealed low standard deviations in FEL content at all compositions (Fig. 3), indicating HME filament homogeneity suitable for subsequent FDM printing.

Target FEL content was 10% w/w, 30% w/w, and 50% w/w FEL for FEL10, FEL30, and FEL50, respectively, from physical mixtures before HME. Although the measured FEL content in HME filaments was lower than the target FEL content for all compositions (Fig. 3), it is evident that the filaments from the second extrusion have a higher FEL content (86–88% of target content) than the filaments from the first extrusion (74–78% of target content) at all compositions. Due to incomplete emptying of the barrel between compositions despite mechanical cleaning, this was largely attributed to carry-over of residue containing both drug and polymer from the preceding extruded composition of lower FEL content. The below-target FEL contents were a consequence of both incomplete emptying of the barrel between compositions and the order in which the compositions were extruded (from lowest to highest FEL content) following an initial priming of the extruder with pure EC, resulting in dilution of each subsequent extruded composition as residual material was replenished with incoming material. Residual material in the extruder barrel and on the screws would be a much less pronounced issue in a scaled-up process when equipment with a smaller effective surface area per unit volume, screw designs suited to optimal conveying, and continuous feeding of powder feedstocks to maintain a constant barrel pressure, are implemented.

**Fig. 4:**
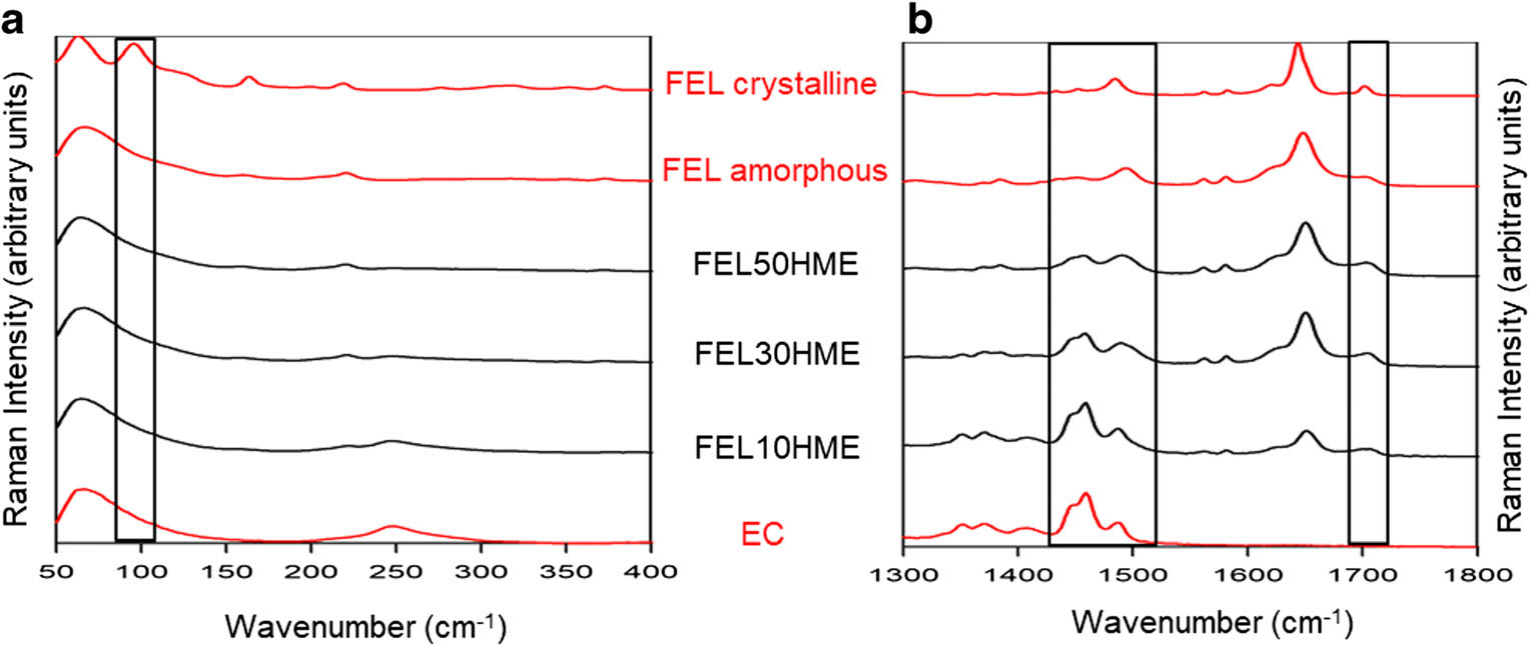
Raman spectra of raw materials and HME filaments in (**a**) the lattice mode region and (**b**) the fingerprint region.

Since the HME and FDM processes are run in batch mode via two distinct unit processes, acquired homogeneity after HME must be maintained in the storage period prior to FDM printing, which was investigated and confirmed using FT Raman spectroscopy and DSC. Figure 4 shows Raman spectra of raw materials and HME filaments in the lattice mode region (Fig. 4a) and the fingerprint region (Fig. 4b) measured 2 weeks after HME. The lattice mode region of the Raman spectrum allows rapid discrimination between solid-state forms and could therefore be used to denote process-induced changes in the solid-state form of FEL thereby confirming an absence of recrystallization after storage at ambient conditions prior to FDM.

**Fig. 5:**
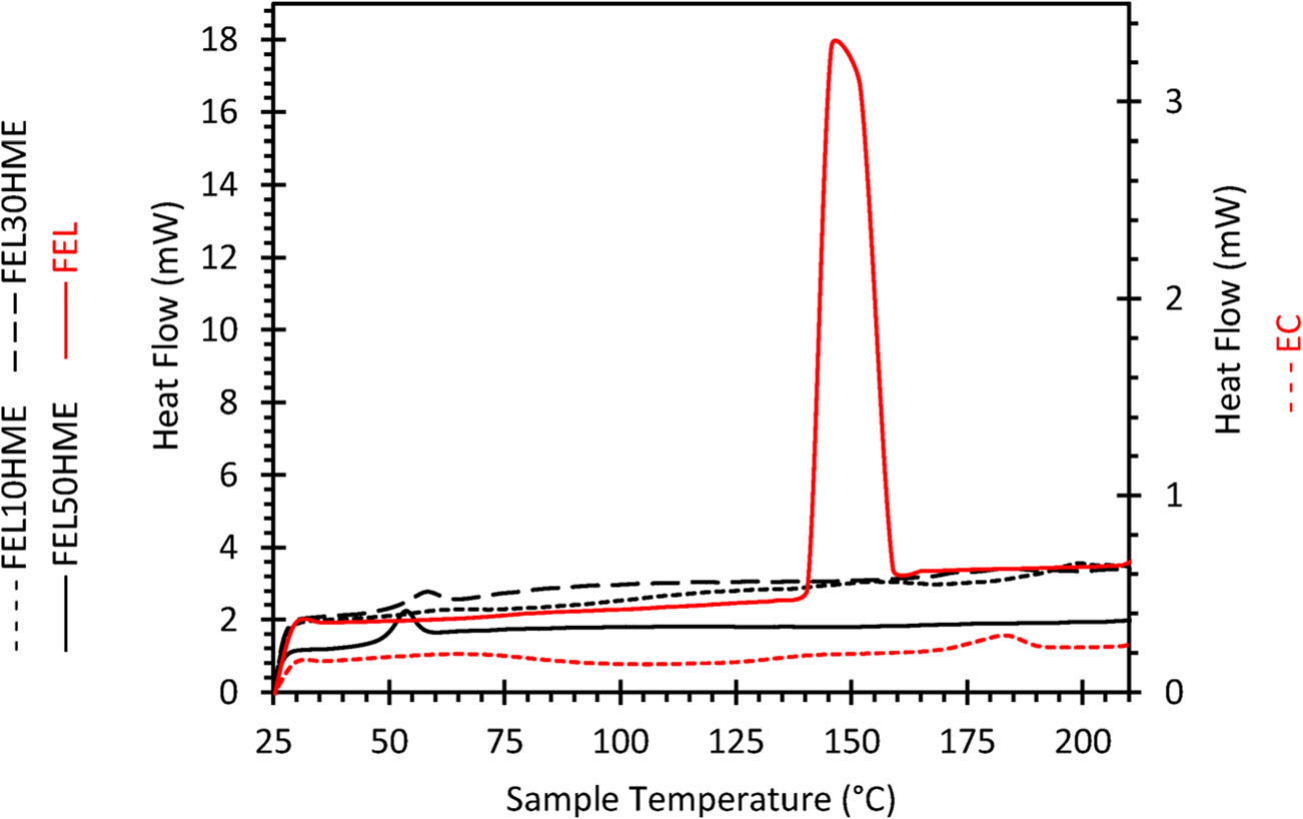
DSC traces of extruded filaments (black lines) relative to raw materials (red lines) for heat cycle 1.

Crystalline FEL displays a characteristic sharp band at 96 cm^−1^ in accordance with the literature (30), which is not present in the amorphous FEL or in the extruded filaments (Fig. 4a). The fingerprint region of crystalline FEL powder also reveals a crystalline FEL band at 1701 cm^−1^ corresponding to the methyl ester carbonyl of FEL (31) (Fig. 4b). Whilst this peak is present in amorphous FEL and in HME filaments, it is broader and poorly resolved from the adjacent peak (30). This indicates an absence of FEL recrystallization in extruded filaments prior to FDM. Furthermore, the relative intensity of the amorphous FEL peak at 1494 cm^−1^ to the EC peak at 1459 cm^−1^ increases as FEL content in the filament increases, indicating that the acquired spectra are representative of expected FEL contents in the bulk filament. Taken together, the fingerprint region confirms the presence of FEL in expected proportions to the carrier in all extruded filaments and the lattice mode region confirms process-induced changes in FEL from crystalline to amorphous. This result is in agreement with the absence of FEL melting endotherms in the first heat cycle of DSC of extruded filaments (Fig. 5).

The peaks at 60°C and 54°C for FEL30HME and FEL50HME, respectively, overlay the T_g_ and correspond to molecular relaxation upon heating above T_g_ as a consequence of relieving extrusion-induced stresses present in these filaments (Fig. 5) (32).

If FEL aggregates were present in our system at high FEL contents, they were amorphous and not detectable as a second T_g_ during DSC, but more significantly, they did not hinder obtaining mean FEL content within 5% of target values. The formation of solid solutions is not a strict requirement for accurate and precise flexible dosing but, in accordance with our hypothesis, the extent of homogeneity, given by the size and distribution of aggregates, must be appropriate for the desired print volume.

### Dispensing Precision

The initial processing window was determined from the thermal properties of the raw materials as measured by TGA and DSC on raw materials. The upper limit for processing temperatures during HME was defined by the T_deg_ whereas the lower limit was defined by the T_g_ and T_m_, as reported in Table I. It is recommended that HME be performed 20–40°C above T_g_ to maintain the polymer in a rubbery state at a suitable melt viscosity for extrusion. Based on the results in Table I, an extrusion temperature of 150°C was found to be suitable, which would also allow melting of crystalline FEL (33).

**Table I:** Onset of Thermal Degradation T_deg_ (°C), Glass Transition Temperature T_g_ (°C) and Melting Point T_m_ (°C) of Raw Materials

Material	T_deg_ (°C)	T_g_ (°C)	T_m_ (°C)
FEL powder	287	~45	148
EC powder	300	~128	185

**Fig. 6:**
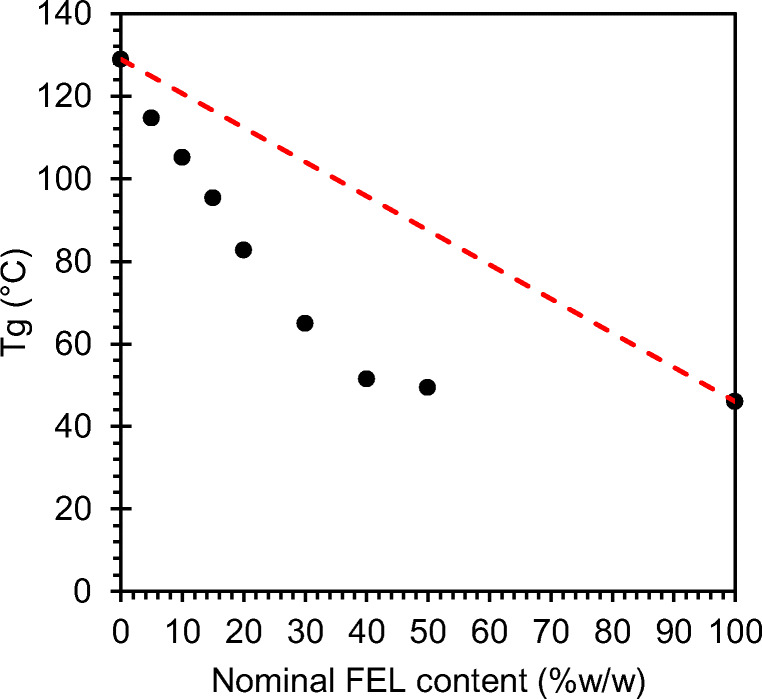
Tg *vs.* nominal FEL content (% w/w) in HME filaments. Dashed red line denotes linear Gordon-Taylor model. Pure FEL Tg determination was performed on powder due to an inability to extrude pure drug.

Precise dispensing requires consistent melt flow from the FDM nozzle. To achieve this, FDM is generally performed at temperatures higher than HME to compensate for the relatively short residence time of the filament in the FDM liquefier, however, processing temperatures must also account for the potential influence of the drug on melt flow at different compositions. To determine the required adjustment of processing temperature for FDM, T_g_ of extruded filaments of varying compositions was determined to investigate the influence of FEL on the T_g_ of the binary mixture of FEL and EC during processing. The Gordon-Taylor Eq. (1) is a model widely used to explain the composition dependence of the T_g_ in amorphous binary mixtures, *e.g.* plasticization and anti-plasticization (34). The T_g mix_ of the mixture is derived from contributions of the pure components (T_gi_) and is defined as

1 $$ {T}_{g\  mix}\approx \left[{\omega}_1.{T}_{g1}+K.{\omega}_2.{T}_{g2}\right]/\left[{\omega}_1+K.{\omega}_2\right] $$

where ω_1_ and ω_2_ are the mass fractions of each component, T_g1_ and T_g2_ are their respective glass transition temperatures, and the constant K is an adjustable fitting parameter. Notably, the Gordon-Taylor equation is based on volume additivity and assumes ideal volume mixing of both components in the absence of specific interactions, resulting in linearity of the dashed line in Fig. 6.

Experimentally obtained T_g_ values display a convex downward deviation from the linear Gordon-Taylor model (Fig. 6). This nonlinear decrease in T_g_ as a function of filament composition indicates that the T_g_ of FEL predominates in intensity in the binary mixture and confirms plasticization of EC by FEL. It may be attributable to intermolecular forces between FEL and EC, which manifest as specific interactions between functional groups of FEL and EC, however, this cannot be inferred from deviations from Gordon-Taylor alone (27,28,34,35). Nevertheless, such deviations have been demonstrated previously for FEL in cellulosic carriers (36). Interestingly, at low FEL contents, T_g_ of the mixture decreases approximately in proportion to the weight fraction of the lower T_g_ component, FEL, which may indicate a well-mixed system until the levelling out of T_g_ beyond 50% w/w FEL, which may suggest phase separation into a second FEL-rich phase as one or more FEL aggregates. Whilst this is in agreement with previous observations for mixtures of FEL and HPMC or HPMCAS (36,37), as indicated in section 3.1, it did not impact attaining target FEL contents.

**Fig. 7:**
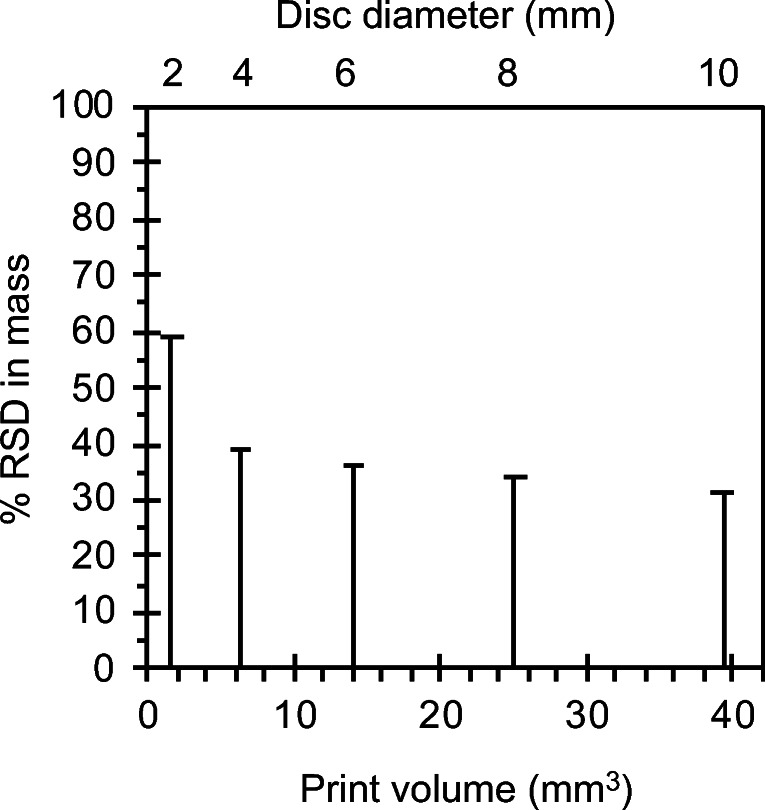
% Relative standard deviation (RSD) in mean mass *vs.* print volume (mm3), *n* ≥ 27.

Despite modification of FDM printing temperatures to account for plasticization of EC by FEL, large relative standard deviations in mean mass reveal poor dispensing precision (Fig. 7).

This reflects variations in disc thickness at the level of a single disc for all print volumes in this study. Since filament brittleness prevented automated feeding, this dispensing imprecision likely signals a sensitivity to manual feeding rates (38,39). However, mass deviations revealing over-extrusion and under-extrusion from the FDM nozzle were encountered previously, despite automated feeding, at both larger print volumes (74 to 468 mm^3^) (21) and smaller print volumes (20). This could, in part, be due to filament diameter variations. Mean filament diameters were 1.54 ± 0.09, 1.49 ± 0.14 and 1.34 ± 0.08 for FEL10HME, FEL30HME and FEL50HME, respectively (*n* ≥ 28 filament sections measured) in our study. Furthermore, the greater imprecision observed at smaller dispensing volumes implies that inconsistent deposition rates of the melt from the FDM nozzle is an additional contributor to dispensing imprecision.

## Discussion

### General Applicability of the High Drug Content Approach in Promoting Flexibility of the FDM Platform

**Fig. 8:**
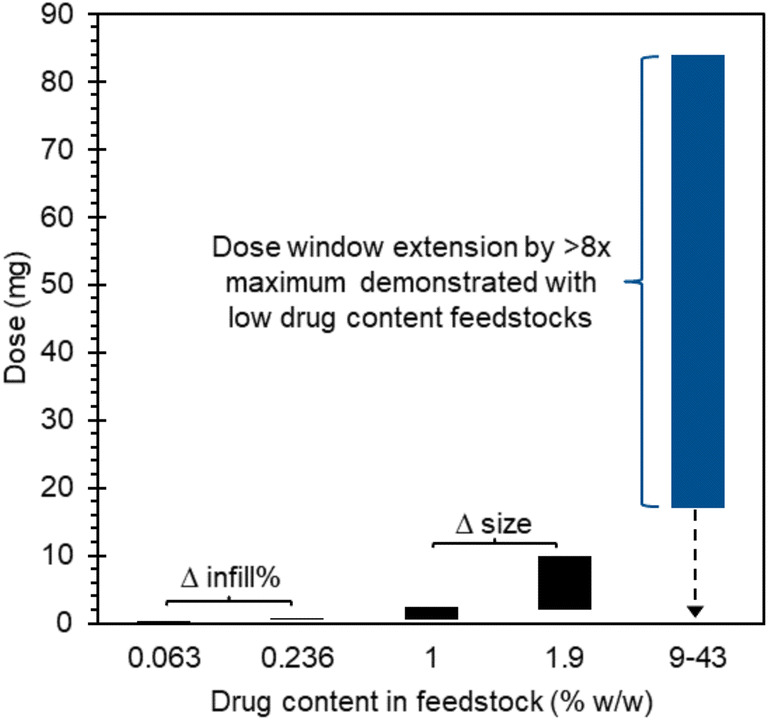
Extension of FDM dose window with the high drug content approach relative to the low drug content approaches based on 0.063% (20), 0.236% (20), 1% (19) and 1.9% (21) w/w API in feedstocks demonstrated in current literature.

Figure 8 depicts the extension of the dose window, relative to that demonstrated by current studies, exemplified for a standard-sized 200 mg dosage form comprising multiple small volume printed units manufactured using the high drug content approach in this study.

Dose flexibility currently demonstrated by FDM, involving the use of comparatively lower drug content feedstocks, are depicted for comparison (19–21). A greater than eight-fold extension of the upper limit of the dose window, relative to the maximum dose demonstrated in current literature, has been achieved in this study. The dose range for a 200 mg dosage form was calculated based on mean measured drug content in the printed discs of 8.6, 25, and 41.8% w/w FEL for FEL10FDM, FEL30FDM, and FEL50FDM, respectively. The dotted arrow signifies that a lower dose range is accessible via the use of lower feedstock drug contents or incorporation of placebo modules for modular product design concepts.

**Fig. 9:**
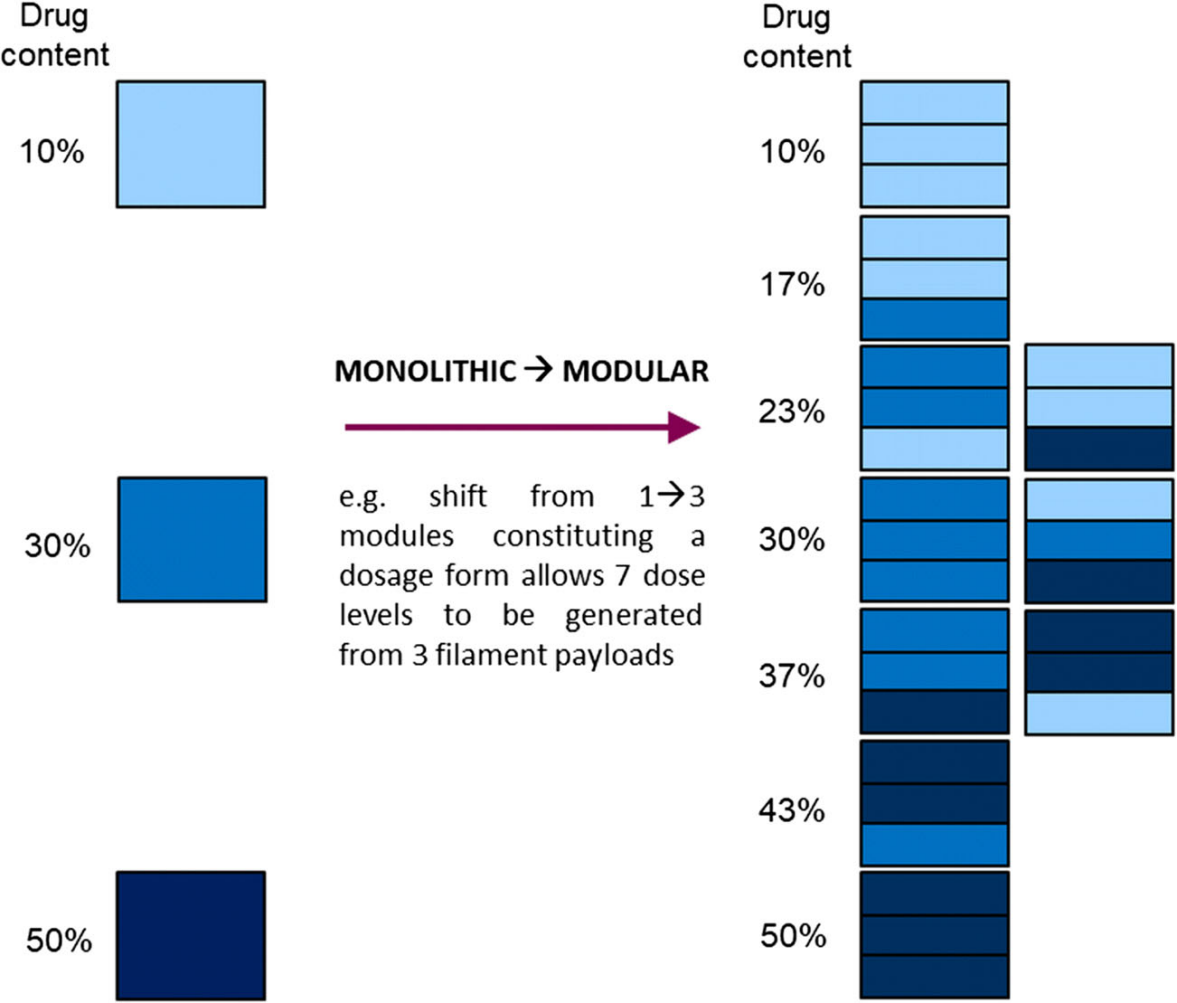
Modular dosage form design concept to enhance dose flexibility.

To enhance dose flexibility within the extended dose range enabled by the use of high drug content feedstocks, we suggest that combining a limited number of discrete feedstock compositions, spanning a wide range of drug contents, could provide a several-fold increase in the number of dose levels for a dosage form. This transition from a monolithic to modular dosage form design concept is workable via the use of multiple nozzles during FDM whilst allowing an increased number of dose levels to be generated from relatively fewer starting filament compositions (Fig. 9), thereby enhancing dose flexibility without a need to generate a designated filament for each desired dose strength.

As depicted in Fig. 9, maintaining acceptable dosage form sizes independent of the dose strength requires printed modules which are a fraction of the size of a conventional dosage form. Since the success of this approach relies on maintaining homogeneity in the feedstock at an equivalent or smaller length scale than the size of a printed module rather than the size of a final dosage form, a modular approach imposes stricter requirements on feedstock homogeneity than the generation of conventional dosage form monoliths. By demonstrating individual drug contents within 10% of the target and mean drug contents within 5% of the target for drug contents up to 42.8% w/w FEL at print volumes relatively smaller than a conventional dosage form, this study has not only confirmed the feasibility of the high drug content approach but notably, can preserve FDM design flexibility by maintaining applicability to a broad range of APIs of varying potencies at conventional dosage form sizes. By extension, for accurate, tailored dosing, the higher the content and smaller the volume that maintains both uniformity of drug content and mass, the greater the potential dose flexibility which may be realised with FDM. Modular product concepts can then be realised in one of two ways, namely, by physical assembly of pre-manufactured subunits or by virtual assembly of various feedstocks or designs via FDM’s .gcode print instruction. The former approach would rely on stability against drug recrystallization for the pre-manufactured subunits at their small size when incorporating drugs with poor aqueous solubility.

#### The Relevance of Amorphous Systems

The maximum drug content to form an amorphous solid dispersion with the desired degree of homogeneity will vary for different APIs and different carriers depending on factors such as drug-polymer solubility, stability against recrystallization, storage conditions, and so forth. In this study, the maintenance of FEL in amorphous form after production as solid dispersions has been confirmed with FT-Raman spectroscopy and DSC. However, although we have considered recrystallization a qualitative indicator of large domain inhomogeneity, neither the formation of solid solutions nor maintenance of the drug in amorphous form are strict prerequisites for dose flexibility. In non-molecularly dispersed systems, the smallest printable module and thus the number of dose increments and degree of dose flexibility will depend upon the size and distribution of aggregates in the system. For the sole purpose of dose flexibility, stable crystalline systems or aggregated amorphous systems are also acceptable, however, clinical relevance for poorly soluble APIs in the case of the former, especially at low potencies, may not be optimal. Crystalline dispersions of poorly aqueous soluble drugs will require higher doses to obtain an equivalent therapeutic effect relative to amorphous formulations of the same drug. The relatively higher feedstock payload that would be needed to deliver a high dose of a crystalline, poorly aqueous soluble drug may compromise homogeneity, depending on the size and distribution of the crystallites. Consequently, our confirmation of FEL in amorphous form at all drug contents in this study also preserves ideal FDM applicability to APIs with poor aqueous solubility.

### The Role of Dispensing Precision in Enhancing Dose Flexibility

Poor dispensing precision at all print volumes and all drug contents in this study was largely attributed to manual feeding. Consistent, automated feeding is a key requirement for precision dispensing during FDM as well as for future scale-up of the FDM platform. This, in turn, relies upon a filament feedstock with high diameter tolerances and either filament feeding mechanisms adaptable to a wide range of filament properties or restriction to use of suitable materials or optimisation of formulations for the types of feeding mechanisms available. Beyond compatibility with current FDM feeding mechanisms, filaments for FDM are required to simultaneously fulfil a host of thermal, mechanical and rheological properties whilst providing the relevant pharmaceutical function (38).

In addition to feeding into the nozzle without filament breakage or deformation, in the absence of wide temperature processing windows, materials of variable rheological properties need to be accurately and consistently dispensed from the nozzle, which necessitates novel engineering solutions for both nozzle and feeding mechanism design.

A key advantage of modularized dosage form designs for scalable dosing is that a designated filament need not be produced for each delivered dose, which is potentially beneficial regarding HME-FDM production process efficiency and costs. The uniformity of drug contents demonstrated in this study fully support this approach, however, until a superior level of dispensing precision can be established on a single module level (at sub-dosage form sizes), achieving scalable dosing via modularized designs cannot be realised yet.

## Conclusions

Extension of the upper limit of the dose window by more than eight times the maximum dose encountered in existing literature has been demonstrated in this study via the use of high drug content solid dispersions, which is a prerequisite for pharmaceutically relevant dose flexibility. The versatility and design flexibility of FDM remains at the core of its potential for personalized pharmaceuticals. Therefore, in an effort to customize product features like the dose strength, FDM multifunctionality must not be compromised. To harness this advantage to its full extent, unlike currently demonstrated FDM dose flexibility studies, which require high potencies and/or large dosage forms for clinically relevant doses, our results support preserved design flexibility via applicability to a broad range of APIs of different potencies and solubilities.

The current work has demonstrated mean drug content within ±5% of target values for all compositions and print volumes but poor dispensing precision at all print volumes. This pertinent result serves to highlight that whilst appropriate material selection may favour the generation of stable, homogeneous, high drug content solid dispersions, dose flexibility cannot be fully realised if the same materials do not exhibit appropriate thermal, rheological and mechanical properties for compatibility with FDM feeding mechanisms and nozzle designs. Generating functional, fit-for-purpose dosage forms has always relied upon material properties appropriate to both processability and the delivery of desired functionalities, however, for the purposes of promoting FDM flexibility, doing so without severely restricting the range of materials that can be used, remains crucial.

### ACKNOWLEDGMENTS AND DISCLOSURES

The authors would like to thank the Nordic POP (Patient Oriented Products) programme for supplying the ZMorph FDM printer used in this study. We would also like to thank Anders Borde (AstraZeneca) for his assistance with UPLC and UV, Johan Arnehed (AstraZeneca) and Hanna Matic (AstraZeneca) for their insights relating to Raman, and Ulrika Thune (AstraZeneca) for sharing her expertise on felodipine. The authors declare that they have no conflict of interest.

## Data Availability

The datasets generated and/or analysed during the current study are available from the corresponding author on reasonable request.

## ABBREVIATIONS

AM: Additive manufacturing
API: Active pharmaceutical ingredient
DSC: Differential scanning calorimetry
EC: Ethyl cellulose
FDM: Fused deposition modelling
FEL: Felodipine
HME: Hot melt extrusion
TGA: Thermogravimetric analysis
